# ERRATUM: Assessment of workload in the postoperative period of cardiac surgery according to the Nursing Activities Score

**DOI:** 10.1590/1980-220X-REEUSP-2024-ER11en

**Published:** 2024-09-27

In the article “Assessment of workload in the postoperative period of cardiac surgery according to the Nursing Activities Score”, with the DOI: https://doi.org/10.1590/S0080-623420150000700012, published by the journal: “Revista da Escola de Enfermagem da USP, volume 49 (spe) of 2015, p. 79–85, on page 85:

**Now read:**

## Financial support

This study was financed in part by the Coordenação de Aperfeiçoamento de Pessoal de Nível Superior – Brasil (CAPES) – Finance Code 001.

