# Supplementary material for: ERRATUM: Assessment of workload in the postoperative period of cardiac surgery according to the Nursing Activities Score
Source: Rev Esc Enferm USP. 2024 Sep 27;58:e2024ER11. doi: 10.1590/1980-220X-REEUSP-2024-ER11en (PMC11441792; doi:10.1590/1980-220X-REEUSP-2024-ER11en)
Supplement: Translation

# Translation: ERRATA: Avaliação da carga de trabalho no pós-operatório de cirurgia cardíaca segundo o Nursing Activities Score

No artigo “Avaliação da carga de trabalho no pós-operatório de cirurgia cardíaca segundo o Nursing Activities Score”, com número de: DOI: https://doi.org/10.1590/S0080-623420150000700012, publicado na Revista da Escola de Enfermagem da USP, volume 49 (Esp) de 2015, p. 80–86, na página 86:

**Leia-se:**

## Apoio financeiro

O presente trabalho foi realizado com apoio da Coordenação e Aperfeiçoamento de Pessoal de Nível Superior – Brasil (CAPES) – Código de Financiamento 001.
